# Intelligent identification of rock mass structural plane and stability analysis of rock slope block

**DOI:** 10.1038/s41598-022-18171-2

**Published:** 2022-10-06

**Authors:** Jinhou Zhang

**Affiliations:** Xi’an University of Science and Technology, Xi’an, 710054 China

**Keywords:** Environmental sciences, Geology, Petrology, Tectonics

## Abstract

The blocking problem caused by structural plane cutting is often the primary problem in evaluating the stability of rock mass engineering. The geometric parameters of the block structural plane are important research bases for analyzing block stability. In order to solve the problems of low efficiency, heavy workload, and high subjectivity in the traditional acquisition of structural plane occurrence information, taking the slope at the entrance of Suichang Gold Mine National Mine Park as the research background, this paper studies the rapid extraction method of structural plane information based on three-dimensional laser scanning data, and puts forward the process and data processing method of using a 3D laser scanner to collect slope block structural information to obtain point cloud data. Based on the coordinate projection principle and MATLAB, the rock slope block stability analysis CPG program was developed, which realized the whole process from data acquisition to block stability analysis. The research results are essential for identifying structural plane and block stability analysis and can provide theoretical and technical support for practical engineering applications.

## Introduction

The rock mass is composed of structural planes and their surrounding structures. Because of the randomness and irregularity of structural planes, rock mass structure is diverse. The distribution and combination characteristics of structural planes in rock mass determine the engineering geological properties and mechanical properties of rock mass and constitute an important control factor for geological engineering problems of various rock masses. Therefore, the study of rock mass structure and accurate description of its properties have always been a hot and challenging topic in engineering geology and rock mechanics^1^.

There are many block geometry and stability analysis methods at home and abroad, given the above problems. For example, Shi Genhua proposed the block discontinuous deformation analysis (DDA) method based on the discontinuity of rock mass medium, taking the role of joints into account in the rock mass stability analysis, and finally proposed the block theory^2^. Shahami et al.^3^ studied the influence of external load on the displacement of rock blocks by the DDA method. It is concluded that the influence of external force on the stability of block rock mass depends not only on the size of the applied force but also on the size of the block. Johari^4,5^ studied applying the Joint Distributed Random Variables (JDRVs) method in probabilistic analysis and reliability evaluation of plane sliding rock slope stability. Zheng et al.^6,7^ used vector method to analyze probability block theory and developed relevant programs, indicating the importance and superiority of probability analysis relative to deterministic analysis. Sun et al.^8^ explored the stability of engineering rock mass by using the stereographic projection mapping method. The above research methods on block stability and failure have advantages and disadvantages. On the whole, they need to be studied in many aspects, such as the integrated integration of structural plane and free face data, multi-block problems, and so on. Therefore, Yang et al.^9,10^ proposed the coordinate projection method based on the orthographic projection and absorbing the advantages of the stereographic projection. Gao et al.^11–14^ computerized the coordinate projection method, which greatly improved its efficiency in applying structural plane and block stability analysis. Yuan et al.^15,16^ shows that the coordinate projection mapping method and its computerization in the block slope stability analysis have a good application effect through the example analysis.

The information on the rock mass structural plane is the basis of rock mass structural analysis and rock mass stability evaluation. Before using coordinate projection to analyze block stability, it is necessary to collect fundamental data such as the occurrence of the structural plane. At present, there are four main types of technical methods used to collect structural plane information: traditional compass measurement method^17,18^, borehole directional coring technology and borehole photography technology^19^, photogrammetry method^20–22^, and three-dimensional laser scanning method^23,24^. The compass measurement method is currently the most common and accurate for geologists to obtain structural plane information. However, the workload is large, and the field time is long; it is sometimes difficult to get comprehensive structural plane information due to adverse environmental factors such as weather and terrain. The drilling method^25,26^ requires high drilling quality and cannot reflect the occurrence information of a large structural plane. In addition, the interpretation accuracy of this method cannot be guaranteed. The photogrammetric method^27^ has won the wide attention of many scholars at home and abroad to acquire rock mass structural plane information without contact measurement, comprehensive acquisition of rock mass structural information, strict mathematical formula, and high-precision observation. However, there are many factors affecting the measurement accuracy, such as the stability error of the azimuth element in the digital camera, the residual error of the digital image distortion correction, the external orientation error of the camera, and many factors affected by the working environment, which are not widely used in practical engineering. The 3D laser scanning method^28–30^ has the advantages of non-contact, long-distance, high precision, fast speed, complete digitization, and visualization, which has incomparable advantages over traditional survey methods and opens up a new way for rapid acquisition of block boundary conditions in areas with poor geological conditions. For example, Singh et al.^31,32^ proposed different algorithms to extract the discontinuous features of rock mass based on the 3D laser scanning method. Azarafzac^33^ used image processing techniques to extract rock mass features to identify blocks.

Therefore, this paper takes Suichang Gold Mine National Mine Park as the research background, uses a 3D laser scanner to collect data, proposes the process and pretreatment method of using a 3D laser scanner to collect slope block structure information to obtain point cloud data, and compares the measurement results with the line measurement method. Combining the advantages of high efficiency and accuracy of the data collected by the new measurement technology with the benefits of coordinate projection in the analysis of block stability, using MATLAB to develop and compile the rock slope block stability analysis program—CPG program, to realize the intelligent identification, information extraction of rock structural plane and block stability analysis, which provides certain theoretical guidance for the combination of geological engineering and surveying and mapping. It has theoretical research significance and has essential practical engineering application value.

## Rock mass structural plane information collection and data processing

The investigation and occurrence analysis of structural planes in the rock mass is one of the basic works for engineering rock mass stability analysis. The traditional data acquisition uses the scanline method, which is influenced by the technical level of the measurement personnel, the measurement environment, the low efficiency, and the large measurement workload. Therefore, this paper uses the rapid development of 3D laser scanning technology to collect data. Since the point cloud data obtained by the 3D laser scanner is polar coordinate data with a large number and many noise points, it cannot be directly used, and it is necessary to process the collected basic data and transform them into the three-dimensional rectangular coordinate data that can be used by the coordinate projection method. Then, combined with the coordinate projection principle, it can determine which structural planes and free surfaces can be combined into blocks, and determine the stability of blocks, to improve the efficiency of block stability analysis.

The distribution of the structural surface of the Suichang gold mine slope is investigated using the scanline method and the 3D laser scanning method, respectively. The measurement results of the two were compared to verify the accuracy of the 3D laser scanner in measuring the structural plane.

### Survey structural plane by scanline method

The scanline method uses the ruler and geological compass as the primary measurement tools to measure the structure plane and collect each structural plane's information. It is the most common and accurate method to obtain structural plane information.

#### Basic slope characteristics

**Figure 1:**
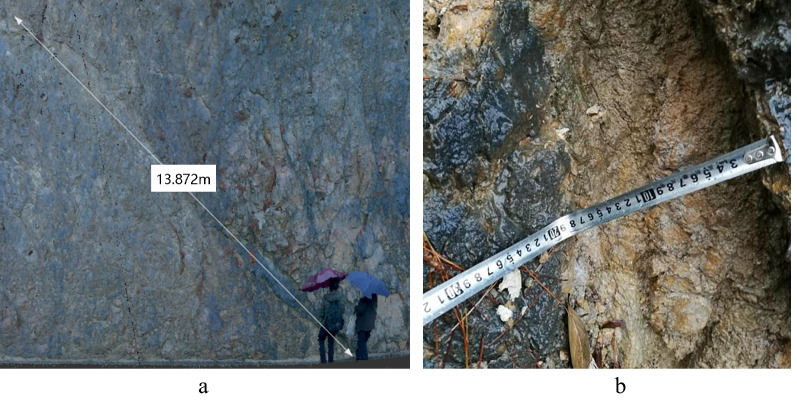
Grade III structural plane J_1_ at 40 m from the inlet. (**a**) Overall appearance of muddy weak interlayer. (**b**) The average width of muddy weak interlayer.

The Suichang Gold Mine National Mine Park is located in Huayuanling, Suichang County, Zhejiang Province. There is a steep rock slope at the ticket entrance waiting position of the scenic park spot and the Panshan highway leading to the central scenic spot. The slope height is between 20 and 23.5 m, and the slope is about 65°. The lithology is biotite plagioclase gneiss. There are more fractured rock blocks in the upper part of the slope and fewer fractured rock blocks in the lower part. The structural planes cut out of rock blocks are primarily in the bedding direction, and the occurrence is similar to the inclination of the slope. There are nine large structural planes on the slope. At 40 m from the inlet, there is a muddy interlayer of about 14 m in length and an average width of about 20 cm, named J_1_, as shown in Fig. 1. J_1_ is a muddy interlayer with good extension, and its bottom width is 40 cm. According to the grade of the Gu Dezhen rock body structural plane^34^, it is classified as a grade III structural plane. On rainy days, the weak interlayer is completely muddy, which is easy to causes a significant influence on the stability of slope rock mass.

In addition, there are many grade IV and grade V structural planes, which are large in number and different in characteristics. The structural planes of this grade mainly affect the mechanical properties of the rock mass on the slope's surface and have a significant influence on the stability of the rock mass, which will not be repeated here.

#### Structural plane information survey

**Figure 2:**
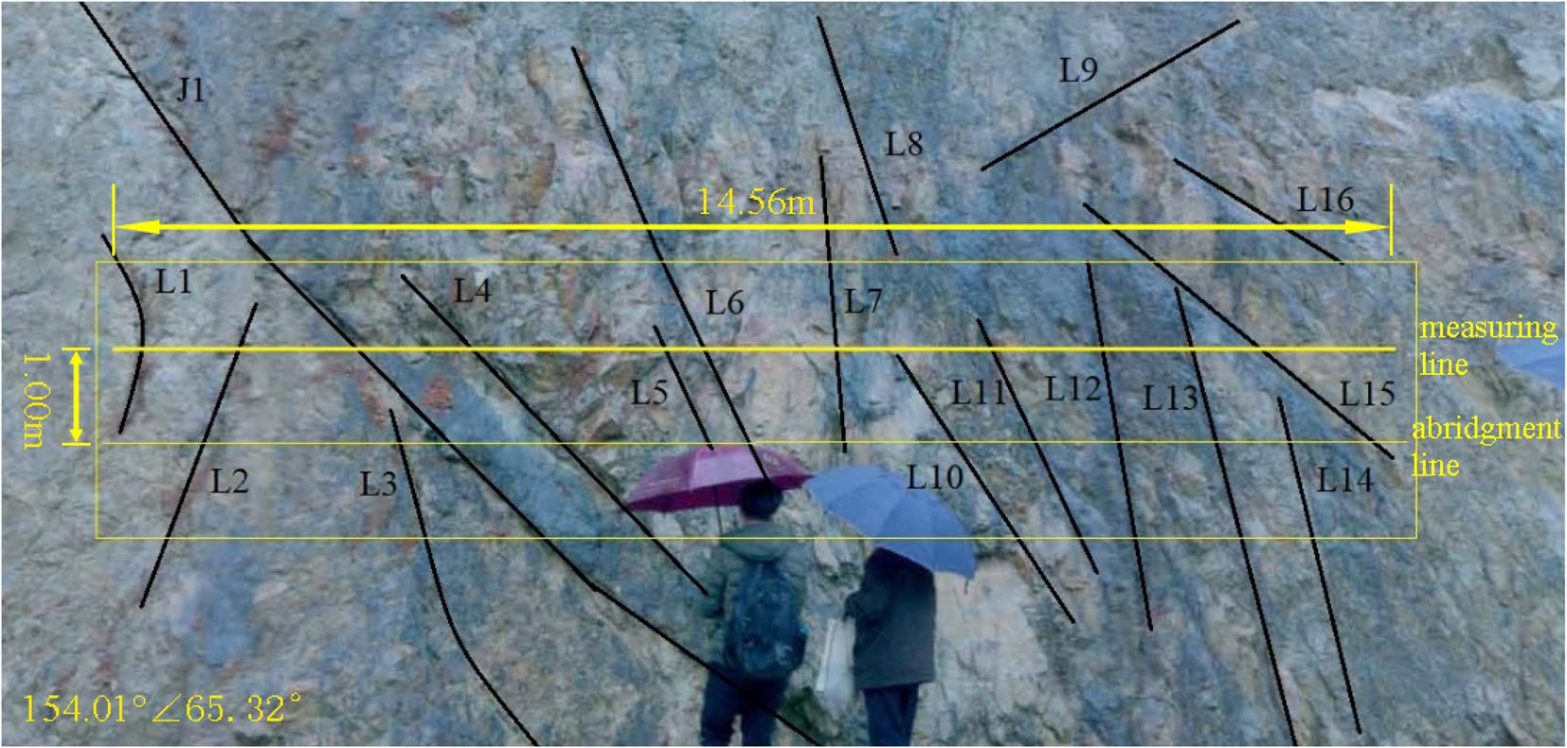
Distribution of rock mass structure plane 40 m away from the entrance of Suichang Gold Mine National Mine Park.

The dense part of the structural plane at 30–45 m from the beginning of the slope is selected as the survey line layout area. The distribution of structural planes in the survey area is shown in Fig. 2. Most of these structural planes are approximately perpendicular to the sloping trend, and the length of structural planes shown in the figure is more than 1.5 m.

**Figure 3:**
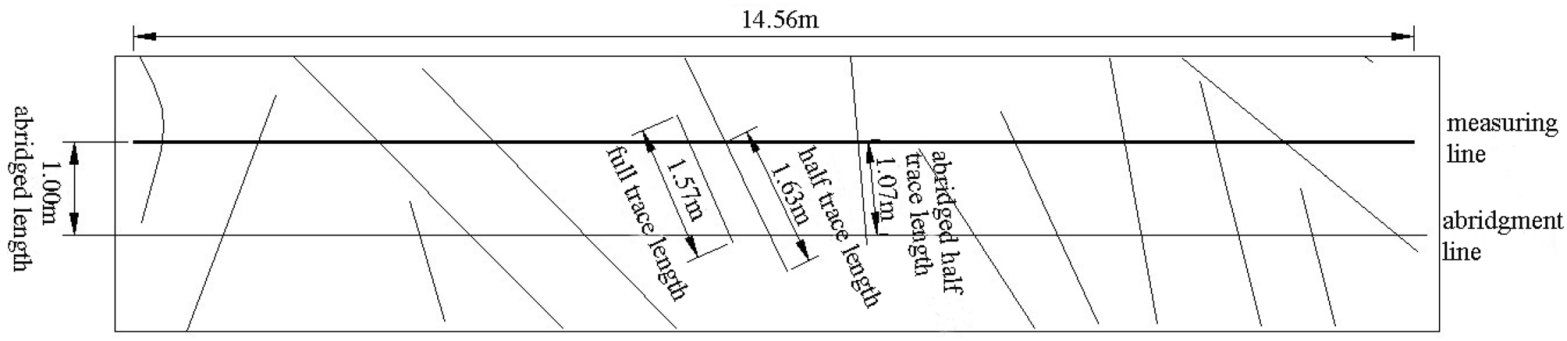
The diagram of the relationship between measuring line and trace line.

It is tough to accurately measure the trace length of the structural plane because the exposed results of the rock mass structural plane are different, and the length and trend of each structural plane are different. Therefore, the half trace length or abridged half trace length are usually measured according to the part of the obvious exposed trace line. The exposed structural plane traces revealed in Fig. 2 are measured and counted. A measuring line with a length of about 14.5 m parallel to the horizontal plane is set from left to right in the middle of the slope. The measuring line passes through more traces. An abridgment line is set at 1 m below the measuring line, which can measure the corresponding structural plane traces in the statistical diagram (Fig. 3). The measuring line and the abridged line intersect with each trace. The angle between the measuring line and the trace is large, and some are close to vertical, with an average angle of about 75°. In the statistical process, some traces may not be able to measure the trace length, and the half trace length and the abridged half trace length can be counted in the measurement.

### Investigation of structural plane by 3D laser scanning

3D laser scanning technology is a high-tech automated scanning technology that uses a laser scanning device system to obtain 3D data on a target surface quickly, automatically, and in real-time. This paper uses a Leica blk360 3D laser scanner for ground data acquisition. The instrument has a variety of scanning methods, such as infrared sensors with thermal imaging, laser, and visible light imaging, which can be measured under various complex conditions.

#### Point cloud data collection

**Figure 4:**
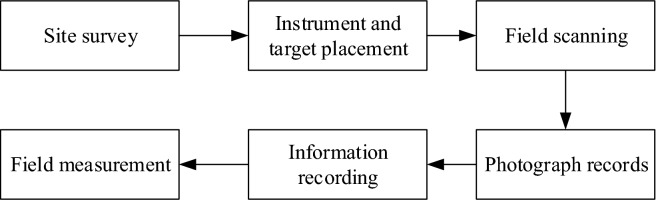
Data collection process.

The specific process of field data collection is shown in Fig. 4. Site reconnaissance aims to understand the surrounding topography and the setting of measurement sites and determine a reasonable layout of scanning measurement sites and targets. After collecting point cloud data, we should use 3D laser scanning digital camera or external camera to collect digital photos to provide auxiliary data for subsequent point cloud data registration. After completing the scanning, some field measurements should be carried out to obtain the block rockfall information of the specific working point and some parameters of the field block structural plane (panorama map, rock identification, structural plane occurrence, spacing, length, filling, and other information), which are convenient for comparison with the late interpretation results of the point cloud.

#### Point cloud data preprocessing

**Figure 5:**
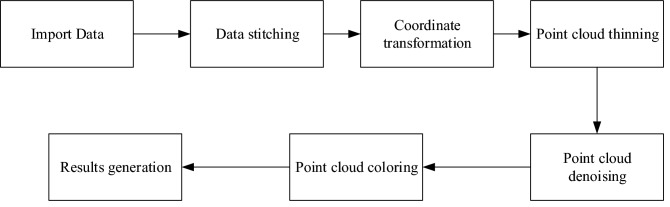
Data processing flow.

The number of point cloud data obtained by scanning is huge, and it contains many miscellanies (such as trees, buildings, etc.), which needs further processing to be used normally. This paper uses Cyclone Register 360 software for data preprocessing, and the primary process is shown in Fig. 5.

1. Data stitching

Point cloud data registration is one of the main steps in point cloud data processing. Due to the limitation of the volume of the target object and the working area, multi-station and multi-directional scanning are usually required. At this time, each station scanning is an independent coordinate system established based on its scanning position. The measured point cloud data are based on the current station's position and the instrument's spatial attitude. Data stitching is the process of restoring the point cloud data of these different stations to the same coordinate system, completing the docking of the point cloud and the determination of the accuracy, and using the same name point to stitch the data to restore the actual scene. Point cloud stitching has three methods: target automatic stitching, pick-up point cloud feature point stitching, and hybrid stitching. Hybrid stitching refers to the combination of target and manual matching feature points.

**Figure 6:**
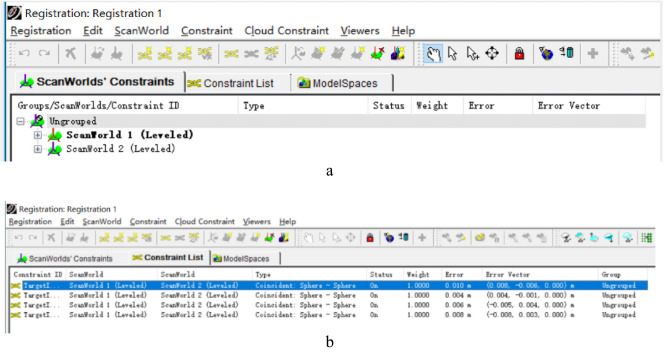
Stitching of point cloud data. (**a**) The setting of the main station and auxiliary station. (**b**) Common feature point error adjustment.

As shown in Fig. 6, this paper uses picking point cloud feature points for stitching. Firstly, establish the Registration Model Space, import the Scan World database of all station data into the spliced Model Space, and set up the main station (the bold font in Fig. 6a); secondly, the point cloud data error threshold of the two stations is limited, and the stitching accuracy at mm level is retained according to the accuracy requirement. Finally, the stitching calculation, freezing stitching, and establishing a new Model Space, point cloud merged into a station.

(2) Coordinate system conversions

**Figure 7:**
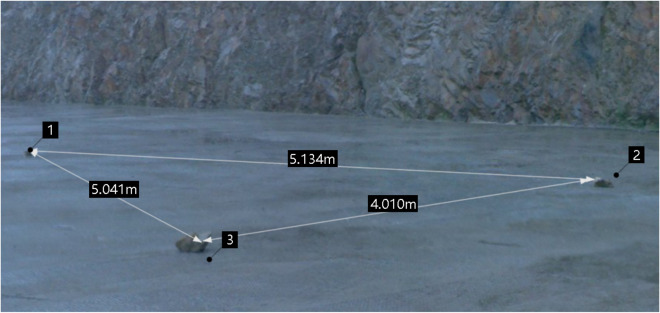
Earth coordinate points scanned by the 3D laser scanner.

After registering point cloud data, the point cloud has a unified coordinate system. However, in practical work, only by converting the scanned three-dimensional coordinates into the absolute geodetic space rectangular coordinate system can the standard data be provided for the production and application of engineering surveys, GIS spatial databases, etc. The main methods and steps are as follows: (1) Finding or laying ground objects with apparent features around any station (Fig. 7 is the ground objects laid in this measurement) so that the 3D laser scanner can scan the ground objects; (2) Taking the geodetic coordinate control point in the Suichang Gold Mine National Mine Park as the benchmark, the geodetic coordinates of ground objects near the station are measured by total station or RTK; (3) Establish a reference coordinate database in Cyclone, and the known coordinates measured by the total station are imported into the reference coordinate database in the form of the point cloud. The column titles of the geodetic coordinate are set as name, x, y, z, and the color matching value RGB; (4) in line with the stitching method of the point cloud, the ground coordinates of the feature points, and the whole point cloud are stitched as a whole so that the point cloud coordinates are unified into the geodetic coordinate system.

(3) Point cloud thinning

The amount of point cloud data after stitching is huge, and the huge amount of data will occupy a large amount of system memory and affect the computer's computing power. Therefore, it is necessary to dilute the point cloud data according to the complexity of the actual project. The point cloud can not be diluted for the essential parts of complex ground objects or structures and the positions that need to be specified. Point clouds without essential structures can be diluted. Finally, complete and uniform point cloud data can be obtained in a geodetic coordinate system. There are mainly the following methods for point cloud simplification: (1) bounding box method; (2) Douglas-Puke thinning algorithm.

(4) Point cloud denoising

When using a 3D laser for scanning operations in the field, the point cloud data obtained from the scan can produce noise due to the complex scanning environment, such as vehicle movement, people walking around, trees, buildings, fog, and floating dust. Before and during the modeling process, it is necessary to continuously check the point cloud and delete these noises to establish a more realistic model as much as possible.

(5) Point cloud coloring

**Figure 8:**
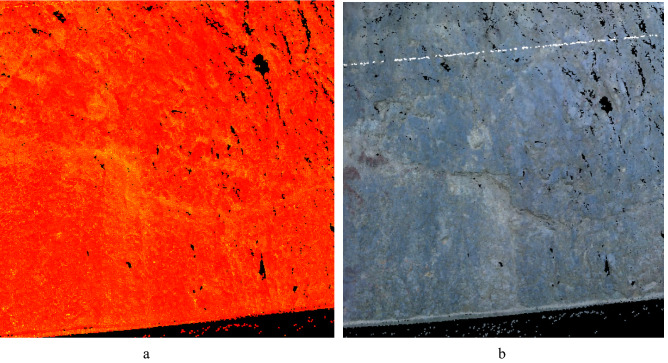
Comparison before and after point cloud coloring. (**a**) Original point cloud data. (**b**) Colored point cloud data.

In order to make the generated 3D point cloud data model the same as the actual target and facilitate modeling and recognition, the color photos obtained by the built-in camera can be mapped to the surface of the point cloud data, and the point cloud data can be manually colored (Fig. 8). Colored point cloud data reflects the color of the actual scanning entity.

**Figure 9:**
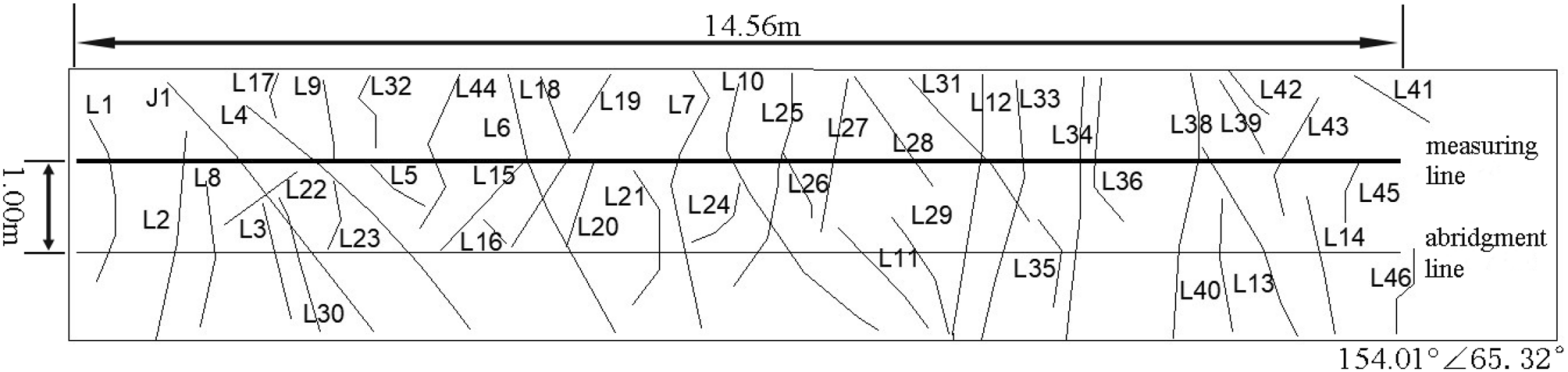
The diagram of the relationship between the scanning line and the trace.

Point cloud data can also measure the trace length and other information. The trace map shown in Fig. 9 can be obtained by connecting the corresponding coordinate points according to the number. Compared with the measurement results of the scanline method, the measurement results of the scanning method are more accurate and can be applied to this project. Due to the more detailed scanning results, the number of scanned traces has increased, and the traces L1, L2, L3, L4, L6, and L12 are numbered in the same way as the manual measurement method, while the other numbered lines are not the same as the manual measurement method.

**Figure 10:**
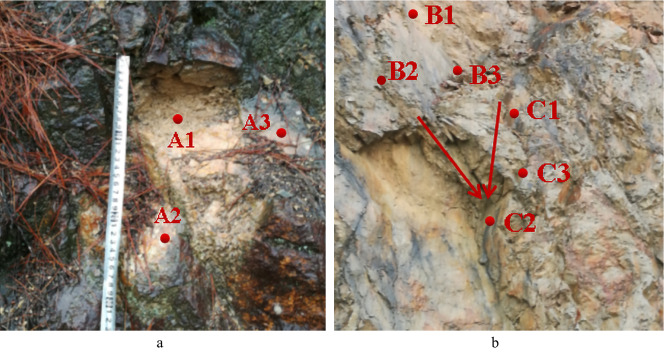
Coordinate points of several typical locations on the slopes of the Suichang Gold Mine Geopark. (**a**) Structural plane of the single-slip surface. (**b**) Structural plane of the double-slip surface.

In order to realize the stability analysis of the block, the coordinate data of the structural plane and the free surface need to be extracted. Figure 10 shows the typical single-slip surface and double-slip surface blocks on the slope. Moreover, the single-slip block has slipped, and the double-slip wedge has not slipped. Measure and output any three-point coordinates on the sliding surface, listed in Table 1.

**Table 1:** Characteristic point coordinates of typical block structural plane.

Point number	X	Y	Z
A_1_	30,344,393.830	10,754,709.153	571.514
A_2_	30,344,394.190	10,754,708.265	570.721
A_3_	30,344,394.514	10,754,709.029	570.710
B_1_	30,344,395.756	10,754,712.496	569.969
B_2_	30,344,395.733	10,754,711.323	569.476
B_3_	30,344,395.681	10,754,711.829	568.993
C_1_	30,344,417.004	10,754,727.803	564.767
C_2_	30,344,395.843	10,754,711.854	568.697
C_3_	30,344,417.004	10,754,727.803	564.767

## Block stability analysis

The usual methods for block stability analysis are analytical, stereographic projection, coordinate projection, and numerical method. Among them, the stereographic projection and numerical methods need to be analyzed by the basic theory of the analytical method. When the possible unstable blocks are identified, and their geometric conditions are determined, the stability of blocks can usually be analyzed by the analytical method. The analytical method is more suitable for analyzing the stability of blocks in slope engineering because of the simplicity of the geometrical and variable force conditions in slope engineering. The coordinate projection method, which is based on the analytical and equatorial projection methods, is more advantageous for the stability analysis of slope blocks.

### Coordination projection method

The coordinate projection method^8^ is a graphical method that combines the orthographic projection and stereographic projection. It can solve the graphic solutions of multiple blocks, curved structural planes, and curves of blocks exposed on the non-flat free surface except for the graphic solutions of a single block and can realize the comprehensive integration of relevant structural plane information. The method is based on the analytical method. It uses the graphical solution to graph the forces acting on the block to determine the stability coefficients of the block and to realize the stability analysis of the block. The graphical method is commonly used in block stability analysis. The orthographic projection method has advantages in length, and the stereographic projection method has advantages in angle. The coordinate projection method combines the advantages of the two methods, expands the application range of the graphic method, and increases the method of block stability analysis. Its primary purpose is to determine the stability of the block by analyzing the geometric relationship between the structural plane and the free surface in rock engineering and analyzing the force system acting on the block.

### Determine the relevant structural planes

The data obtained by 3D laser scanning are the coordinates of the slope body in the whole scanning area. Finding the information of the structural plane from the huge point cloud data is an essential step in obtaining the occurrence. Combining the scanning data with the field geological conditions, such as viewing the site survey records, scanning photos, and artificial photographs. After a comprehensive analysis, the structural plane is fitted according to the point cloud data.

There are two methods for determining the structural plane in the coordinate projection graphical method: the one-point plus attitude equation and the other is the three-point equation. Table 1 shows that the three-point coordinates on the structural plane are not on the same straight line, and the three-point formula can establish the equation. Set on the spatial exposure, the three-point spatial coordinates of a structural plane that is not on a straight line are measured as: $A(X_{1} ,Y_{1} ,Z_{1} )$, $B(X_{2} ,Y_{2} ,Z_{2} )$ and $Z(X_{3} ,Y_{3} ,Z_{3} )$ then the equation of the structural plane can be obtained by the following relevant formulas:

1 $$MX + NY + PZ = Q.$$

In the formula:$M = (Y_{2} - Y_{1} )(Z_{3} - Z_{1} ) - (Y_{3} - Y_{1} )(Z_{2} - Z_{1} ),$

$$N = (Z_{2} - Z_{1} )(X_{3} - X_{1} ) - (Z_{3} - Z_{1} )(X_{2} - X_{1} ),$$

$$P = (X_{2} - X_{1} )(Y_{3} - Y_{1} ) - (X_{3} - X_{1} )(Y_{2} - Y_{1} ),$$

$$Q = MX_{1} + NY_{1} + PZ_{1} .$$

The equation for the structural plane obtained from Table 1 data is $0.89X + 0.01Y - 0.07Z = 27038532$, the trend is $90^\circ + 28^\circ = 118^\circ$, its occurrence is $N28^\circ W$, $SW89^\circ$. The point coordinates of the other two structural planes are substituted into the above formula, and the calculation results are shown in Table 2.

**Table 2:** The occurrence of J_1_ ~ J_3_ structural plane.

Structural plane	J_1_	J_2_	J_3_
Trend	28°	29°	28°
Tendency	*NW*	*SE*	*SE*
Dip angle	89°	27°	12°

### Establishment of horizontal section diagram and block identification

In studying block problems, it is necessary to consider the geometric conditions, size, genetic types seriously, and mechanical properties of the structural planes related to them to better identify them in the field. For this reason, the author makes a horizontal auxiliary plane P through a certain elevation in the three-projection plane system, and then according to the structural plane equation obtained from the above calculation and the position coordinates of the occurrence and points of the structural plane obtained from this, makes the H-plane projection diagram of the intersection line between P plane and each structural plane, and puts forward the horizontal section diagram. The geometric conditions of the structural plane and the free surface of the studied part are comprehensively integrated, and various information about the structural plane and the free surface of the block is systematically studied. Thus, three hexahedrons, namely K_1_, K_2,_ and K_3,_ are determined, and their vertex coordinates are calculated, as shown in Table 3.

**Table 3:** The coordinate values of block vertices obtained from the cross-section diagram.

Coordinate value	A	B	C	D	E	F	G	H	I
X	23.17	22.58	28.63	27.60	23.91	24.08	20.31	18.72	24.17
Y	21.42	23.19	17.30	12.85	9.55	18.08	17.23	19.71	9.23
Z	10.70	5.90	7.75	11.74	11.88	7.91	5.87	9.61	12.91

Coordinate value	J	K	L	M	N	O	P	Q	R
X	21.13	24.58	26.63	27.60	24.91	25.08	20.31	18.72	22.08
Y	21.22	21.19	11.10	14.85	9.52	8.18	17.81	19.78	8.33
Z	10.70	7.90	5.75	10.74	11.88	7.91	5.87	9.61	12.94

### Determination of block interface area

For a general plane block, first, determine the spatial coordinate values of the three vertices on the structural plane that are not on a straight line as $A(X_{a} ,Y_{a} ,Z_{a} )$, $B(X_{b} ,Y_{b} ,Z_{b} )$ and $C(X_{c} ,Y_{c} ,Z_{c} )$, respectively, and obtain the structural plane area by the following formula (2).

2 $$S = \sqrt {p \times (p - a) \times (p - b) \times (p - c)} ,$$

In the formula: $a = \sqrt {(X_{a} - X_{b} )^{2} + (Y_{a} - Y_{b} )^{2} + (Z_{a} - Z_{b} )^{2} } ,$

$$b = \sqrt {(X_{c} - X_{b} )^{2} + (Y_{c} - Y_{b} )^{2} + (Z_{c} - Z_{b} )^{2} } ,$$

$$c = \sqrt {(X_{a} - X_{c} )^{2} + (Y_{a} - Y_{c} )^{2} + (Z_{a} - Z_{c} )^{2} } ,$$

$$p = \frac{1}{2} \times (a + b + c).$$

A series of auxiliary points is determined on the curved block exposure curve. After dividing the plane into several small blocks by dividing the small block area at the interface of the structural plane, the area of each small block surface is calculated according to the formula (2). Finally, the area of the whole surface can be obtained by summation, as shown in Table 4.

**Table 4:** Area of block interfaces.

Block K_1_		Area	Block K_2_		Area	Block K_3_		Area
Interface	J_1_	3.91	Interface	J_1_	6.21	Interface	J_1_	25.27
	J_2_	22.54		J_2_	48.75		J_3_	49.76
	J_4_	19.82		J_4_	22.65		J_4_	58.48
	J_5_	6.20		J_6_	20.31		J_6_	63.44
	J_8_	20.20		J_7_	10.41		J_7_	24.85
	J_9_	21.41		J_8_	43.36		J_8_	43.32

### Solution of block volume

Any plane block can be divided into several tetrahedrons, so the volume of the plane block can be determined by replacing the projection surface elevation method, angle method, and sum vector method. The basic block clipping method can be used to determine the volume of the curved block. Table 5 shows the volume of blocks K_1_, K_2,_ and K_3_. Finally, the coordinate projection graphical method is used to analyze these complex force systems on the block. Then the anti-sliding force and sliding force on the block structure surface are compared to obtain the stability coefficient of the block.

**Table 5:** The volume of each block.

Block name	K_1_	K_2_	K_3_
Volume/m^3^	53.76	49.44	125.22

### Block stability analysis program

**Figure 11:**
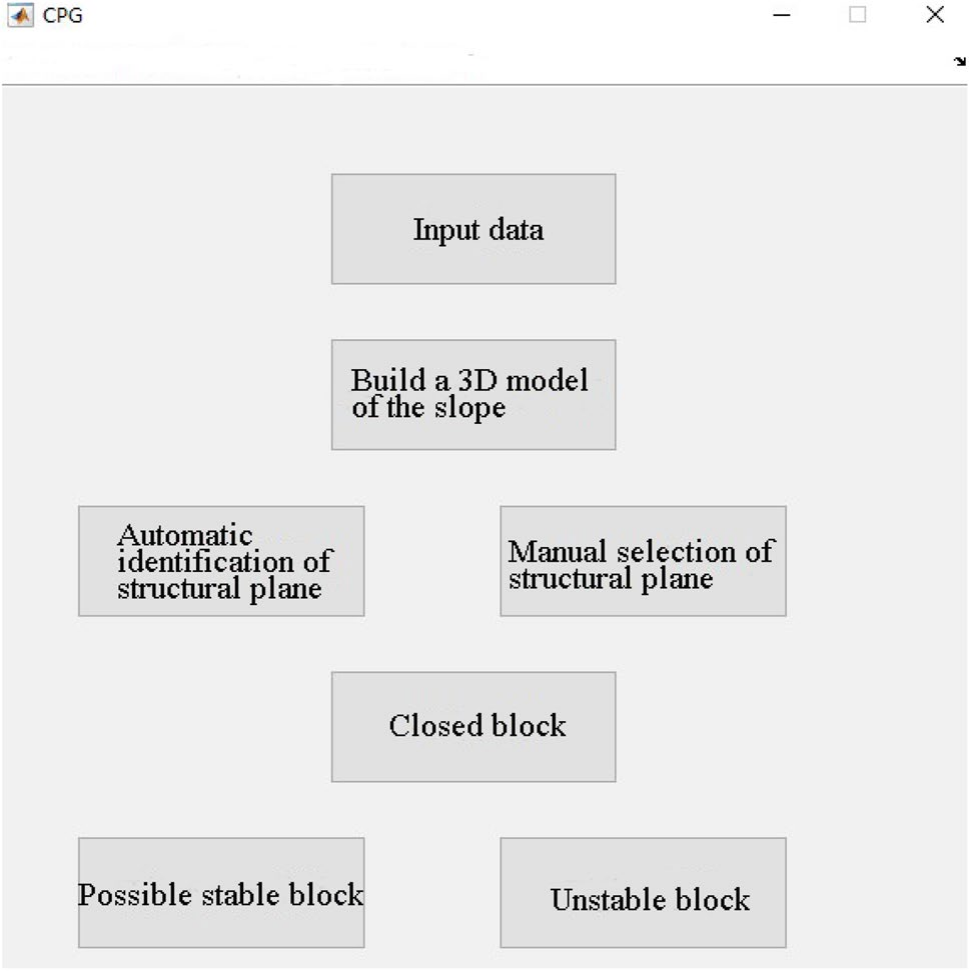
CPG rock slope engineering block stability evaluation program interface.

Since the coordinate projection diagrams of blocks (especially multi-blocks) are more complicated and the drawing efficiency is not high, according to the idea of coordinate projection, combined with the comprehensive and simple MATLAB, the author computerizes it and develops a rock slope block stability analysis program-CPG program (Fig. 11), which can quickly and conveniently determine the geometric block conditions and stability analysis, to improve the efficiency of application in practical engineering.

The CPG program is developed based on the principle of coordinate projection. It can easily form a visual human–computer interaction interface, which greatly simplifies the development steps of the operation interface. At the same time, the image processing toolbox is used for image display, which can truly display the 3D model of the slope and the position of the unstable block. The following five functions can be achieved:

1. Establishment of 3D slope model: CPG-I program

Import the point cloud data obtained by the preprocessed 3D laser scanner and establish the 3D slope model;

(2) Intelligent identification of structural plane: CPG-II program

The primary function is to determine the structural plane equation. This program is divided into two subprograms. The CPG-II-1 program uses the region-growing algorithm in image segmentation technology to automatically search the structural plane from the three-dimensional model. The CPG-II-2 program uses the method of human–computer interaction to allow users to independently select coordinate points from the model to construct the structural plane.

(3) Identification of finite blocks: CPG-III program

The equation of the free surface is constructed. The blocks cut from the structural plane and the free surface are quantitatively analyzed to identify the possible unstable blocks.

(4) Block geometry analysis: CPG-IV program

Calculate the volume of the possibly unstable block and the area of each structural plane;

(5) Block stability analysis: CPG-V program

Calculate the stability coefficient of the possibly unstable block, and finally determine the unstable block.

**Figure 12:**
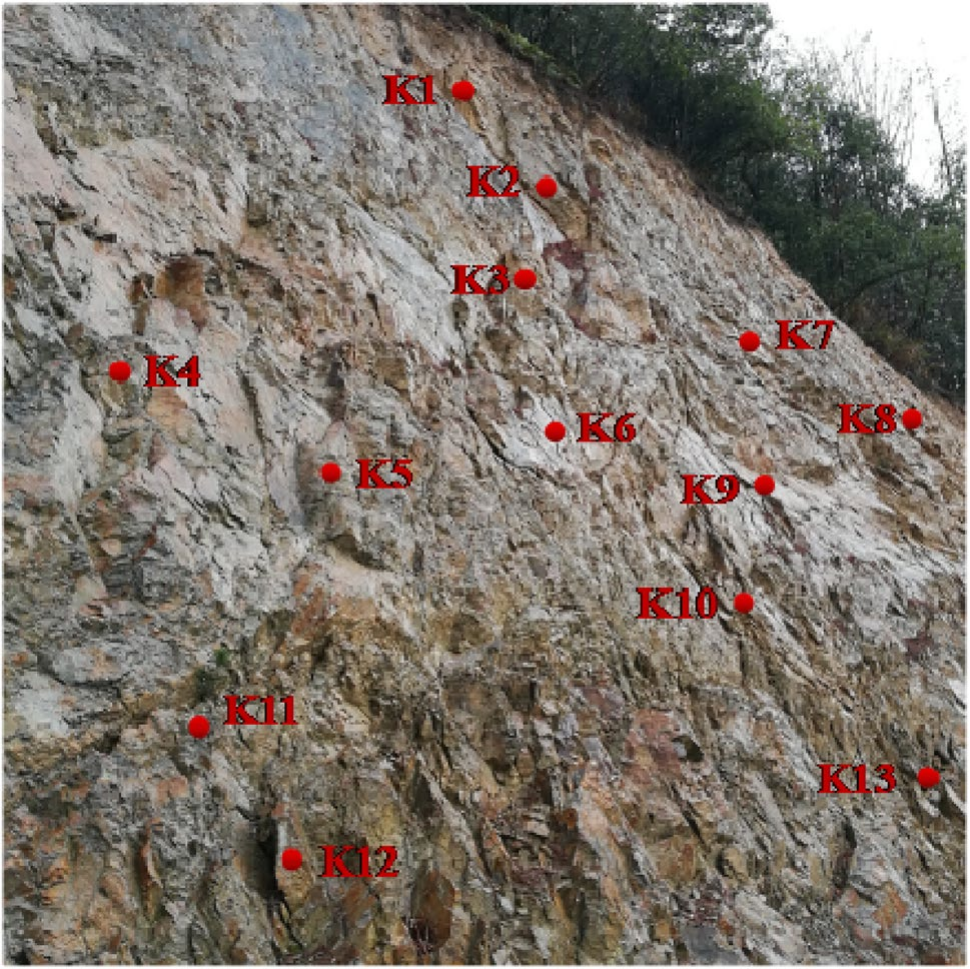
Typical blocks parallel to rock slope surface.

For other typical blocks on the slope (Fig. 12), the point cloud data were first preprocessed using Cyclone software to derive the structural surface information in the form of coordinates and then imported into the CPG program to obtain the stability coefficients listed in Table 6.

**Table 6:** Stability coefficient of each block.

Block name	K_1_	K_2_	K_3_	K_4_	K_5_	K_6_	K_7_
Stability factor	0.66	1.85	2.25	2.13	2.22	2.14	1.35

Block name	K_8_	K_9_	K_10_	K_11_	K_12_	K_13_	
Stability factor	1.1	0.73	1.53	1.62	0.69	0.91	

According to the classification standard of stability degree of dangerous rock mass in Code for geological investigation of landslide prevention (DZ/T0218-2006), it is considered that the stability coefficient of landslide dangerous rock masses is $\eta < 1.0$, unstable block; $1.0 \le \eta < 1.2$, possibly unstable block; when $1.2 \le \eta < 1.3$, the basic stable block; when $1.3 \le \eta$, the stable block. According to this standard, the blocks K_1_, K_8_, K_9_, K_12_, and K_13_ in Table 1 are unstable blocks and need to be treated.

**Figure 13:**
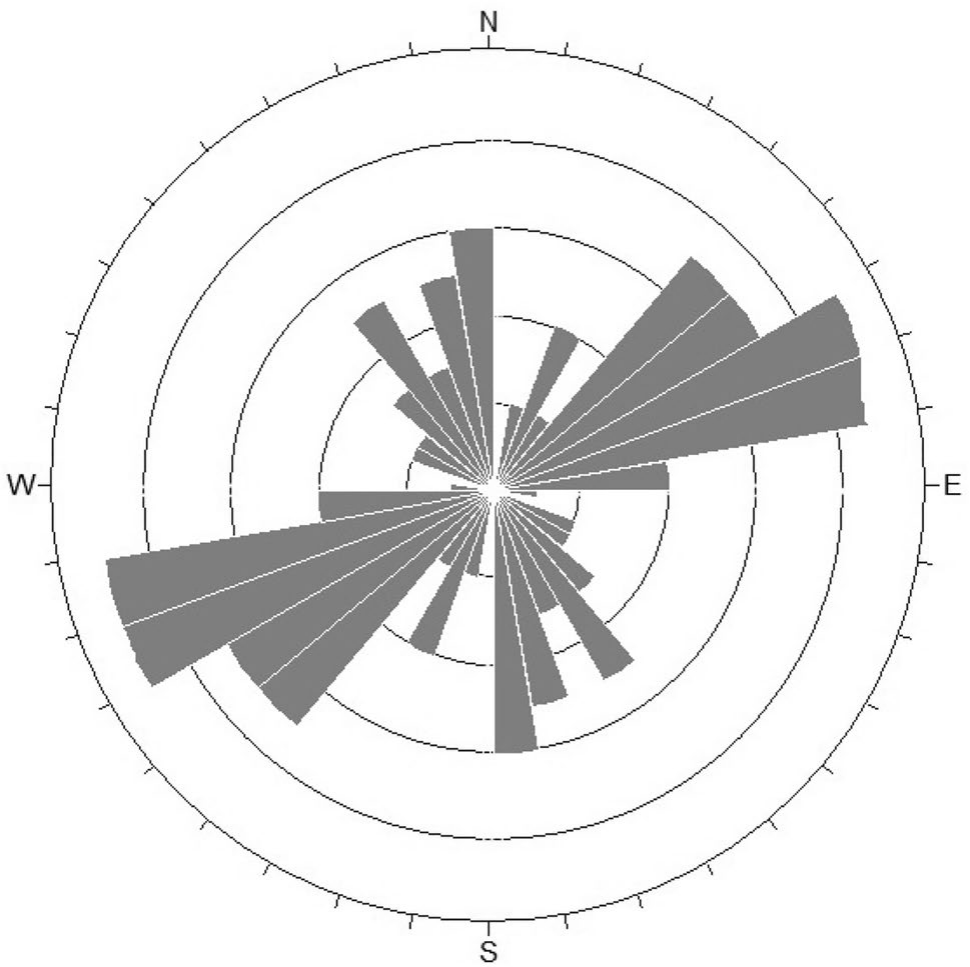
The distribution of deterministic structural plane of slope in Suichang Gold Mine National Mine Park.

The RMR evaluation method is used to evaluate slope rock mass quality (see Table 7). The trend rose diagram of the structural plane in the graphical method is selected as a reference (Fig. 13) to evaluate the geological conditions jointly. According to the RMR value, the rock mass score is 54, belonging to grade III rock mass. The overall condition in general and the local is medium or poor. From the joint rose diagram, the strike of the structural control plane is S70° W, which significantly influences the stability of rock mass. The characteristics of the random structural plane are dense and disorderly. It is basically consistent with the program calculation results.

**Table 7:** RMR classification value of slope rock mass.

	Indicators	Score
Rock strength/MPa	144.7	6
RQD/%	79	17
Joint spacing/cm	20	9
Joint state	The joint surface is slightly rough, the opening degree is less than 1 mm, and the rock on the joint surface is weak	20
Joint condition	Moderation	− 5
Groundwater	Slightly damp	7
RMR	54 (III)

## Conclusion

This paper takes the slope at the entrance of Suichang Gold Mine National Mine Park as the research object, the structural plane of the slope of Suichang Gold Mine is investigated by the scanline method and the three-dimensional laser scanning method, and the Cyclone software is used to process the information collected by the structural plane. Based on the coordinate projection principle, the structural plane equation is determined according to the three-dimensional coordinates of the points obtained after data processing. Finally, the stability of the block is analyzed by the CPG program developed by the authors. The main conclusions are as follows:

1. The rapid acquisition and identification method of rock mass structural plane information is studied. Using a 3D laser scanner to collect the rock mass structural plane's information flow and data processing is proposed. The application in practical engineering and the comparative analysis of the results with the scanline method found that the structural plane data obtained by the three-dimensional laser scanning are more accurate and reliable.
2. The GUI tool in MATLAB was used to develop a program to determine structural surfaces, the automatic identification of blocks, and the discrimination of block stability based on the principle of coordinate projection. The program realizes the functions of importing point cloud data, determining the structure surface of the point cloud (occurrence statistics and plane equation), rock mass information statistics, and automatic identification of block stability.
3. Based on the CPG rock slope engineering block stability evaluation and visualization program, the stability of the typical blocks of the Suichang Gold Mine slope is calculated. The stability coefficients of blocks K_1_, K_8_, K_9_, K_12_, and K_13_ are less than 1.2, which are unstable blocks. Compared with RMR evaluation results, they are basically the same.

## Discussion

The CPG rock slope block stability evaluation program is good at the blocks that have been cut from the free surface or structural plane and has a broad application prospect but does not consider the development of the structural plane. The stability of new blocks may be formed as the structural plane develops or the stability of the remaining blocks after the current block slips.

## Data Availability

All data generated or analysed during this study are included in this published article.
